# Supplementary material for: Secular trends in incidence of type 1 and type 2 diabetes in Hong Kong: A retrospective cohort study
Source: PLoS Med. 2020 Feb 20;17(2):e1003052. doi: 10.1371/journal.pmed.1003052 (PMC7032690; doi:10.1371/journal.pmed.1003052)
Supplement: S1 Table — (DOCX) [file pmed.1003052.s001.docx]

S1 Table: Number of people with incident and prevalent type 1 or type 2 diabetes in the Hong Kong Diabetes Surveillance Database, 2002-2015

|  | 2002 | 2003 | 2004 | 2005 | 2006 | 2007 | 2008 | 2009 | 2010 | 2011 | 2012 | 2013 | 2014 | 2015 |
| --- | --- | --- | --- | --- | --- | --- | --- | --- | --- | --- | --- | --- | --- | --- |
| Number of middle year population of Hong Kong (‘000) | | | | | | | | | | | | | | |
| Men | 3279.6 | 3259.1 | 3266.8 | 3264.0 | 3270.1 | 3283.9 | 3290.2 | 3284.8 | 3294.3 | 3303.0 | 3327.3 | 3329.9 | 3344.5 | 3365.6 |
| Women | 3464.5 | 3471.7 | 3516.7 | 3549.2 | 3587.0 | 3632.4 | 3667.6 | 3688.0 | 3729.9 | 3768.6 | 3822.8 | 3849.0 | 3885.0 | 3925.7 |
| Number of people with incident diabetes | | | | | | | | | | | | | | |
| Type 1 diabetes | | | | | | | | | | | | | | |
| Men |  |  |  |  |  |  |  |  |  |  |  |  |  |  |
| <20 years | 26 | 22 | 14 | 25 | 18 | 28 | 27 | 21 | 24 | 27 | 24 | 24 | 25 | 31 |
| 20-<40 years | 48 | 34 | 32 | 23 | 28 | 35 | 25 | 29 | 19 | 23 | 28 | 31 | 21 | 18 |
| 40-<60 years | 33 | 31 | 32 | 17 | 23 | 16 | 28 | 20 | 23 | 14 | 18 | 18 | 13 | 20 |
| ≥60 years | 20 | 11 | 11 | 8 | 6 | 9 | 4 | 11 | 12 | 6 | 10 | 5 | 13 | 13 |
| Women |  |  |  |  |  |  |  |  |  |  |  |  |  |  |
| <20 years | 34 | 27 | 27 | 29 | 25 | 30 | 38 | 29 | 31 | 26 | 37 | 35 | 34 | 36 |
| 20-<40 years | 47 | 31 | 38 | 42 | 30 | 27 | 25 | 30 | 29 | 18 | 35 | 30 | 36 | 33 |
| 40-<60 years | 25 | 16 | 19 | 18 | 11 | 16 | 11 | 13 | 21 | 21 | 27 | 19 | 15 | 13 |
| ≥60 years | 18 | 5 | 13 | 8 | 9 | 4 | 5 | 11 | 3 | 7 | 13 | 7 | 5 | 9 |
| Type 2 diabetes | | | | | | | | | | | | | | |
| Men |  |  |  |  |  |  |  |  |  |  |  |  |  |  |
| <20 years | 23 | 25 | 30 | 40 | 36 | 43 | 33 | 40 | 48 | 42 | 50 | 49 | 40 | 51 |
| 20-<40 years | 882 | 788 | 935 | 778 | 810 | 897 | 931 | 956 | 983 | 1021 | 1026 | 1061 | 1034 | 1050 |
| 40-<60 years | 6634 | 7241 | 11230 | 7431 | 7360 | 7994 | 8507 | 9291 | 9264 | 10118 | 10090 | 10473 | 9865 | 9732 |
| ≥60 years | 9809 | 10083 | 15820 | 9248 | 8012 | 8511 | 9573 | 10123 | 10784 | 12477 | 11824 | 12023 | 11484 | 11779 |
| Women |  |  |  |  |  |  |  |  |  |  |  |  |  |  |
| <20 years | 35 | 25 | 28 | 50 | 46 | 46 | 43 | 47 | 52 | 51 | 47 | 49 | 60 | 53 |
| 20-<40 years | 658 | 594 | 700 | 590 | 585 | 650 | 669 | 694 | 717 | 763 | 759 | 772 | 802 | 819 |
| 40-<60 years | 5318 | 5984 | 9898 | 6087 | 5847 | 6389 | 7180 | 7493 | 7450 | 8324 | 8195 | 8294 | 7744 | 7933 |
| ≥60 years | 11560 | 12488 | 19734 | 9535 | 8365 | 8849 | 9652 | 10095 | 10573 | 11851 | 11346 | 11477 | 10350 | 10699 |
| Number of people with prevalent type 1 and type 2 diabetes in the middle of the year | | | | | | | | | | | | | | |
| Men | 65959 | 77424 | 99878 | 117219 | 130250 | 142366 | 155598 | 169996 | 184718 | 200948 | 217809 | 233928 | 249265 | 264174 |
| Women | 78519 | 89549 | 114871 | 132726 | 144936 | 155894 | 167881 | 180936 | 194043 | 208039 | 223345 | 237272 | 250615 | 262979 |
| Age-standardized prevalence rate of type 1 and type 2 diabetes* | | | | | | | | | | | | | | |
| Men | 1.6 | 1.8 | 2.3 | 2.6 | 2.9 | 3.0 | 3.2 | 3.4 | 3.6 | 3.8 | 4.0 | 4.2 | 4.3 | 4.5 |
| Women | 1.8 | 2.0 | 2.4 | 2.7 | 2.8 | 2.9 | 3.0 | 3.2 | 3.3 | 3.4 | 3.5 | 3.6 | 3.7 | 4.3 |

*Age standardized to the World Health Organization
