# Supplementary material for: Secular trends in incidence of type 1 and type 2 diabetes in Hong Kong: A retrospective cohort study
Source: PLoS Med. 2020 Feb 20;17(2):e1003052. doi: 10.1371/journal.pmed.1003052 (PMC7032690; doi:10.1371/journal.pmed.1003052)
Supplement: S2 Table — (DOCX) [file pmed.1003052.s002.docx]

S2 Table: Demographic and clinical characteristics of people with incident type 1 or type 2 diabetes at diagnosis in the Hong Kong Diabetes Surveillance Database, 2002-2015

| Number | 562,022 |
| --- | --- |
| Age, years | 61.8 ± 13.2 |
| Age categories, number (%) |  |
| <20 years | 1,956 (0.4) |
| 20-<40 years | 23,769 (4.2) |
| ≥40 years | 536,297 (95.4) |
| Women, number (%) | 270,282 (48.1) |
| HbA1c at diagnosis, % | 7.2 ± 1.1 |
| Fasting plasma glucose at diagnosis, mmol/L | 7.4 ± 1.8 |
| LDL-cholesterol, mmol/L | 2.7 ± 0.7 |
| Triglyceride, mmol/L | 1.4 (0.9) |
| Use of anti-diabetic drugs in first year of diagnosis, number (%) |  |
| Metformin | 372,646 (66.3) |
| Sulphonylurea | 271,721 (48.3) |
| Dipeptidyl peptidase-4 inhibitors | 20,073 (3.6) |
| Thiazolidinediones | 9,403 (1.7) |
| Acarbose | 6,160 (1.1) |
| Insulin | 89,871 (16.0) |
