# Supplementary figures and images for: Secular trends in incidence of type 1 and type 2 diabetes in Hong Kong: A retrospective cohort study
Source: PLoS Med. 2020 Feb 20;17(2):e1003052. doi: 10.1371/journal.pmed.1003052 (PMC7032690; doi:10.1371/journal.pmed.1003052)

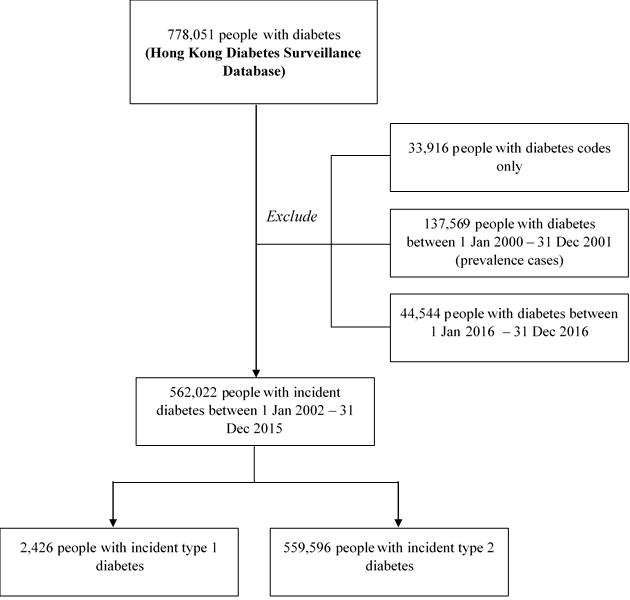

Supplement: S1 Fig — (TIF) [file pmed.1003052.s003.tif]

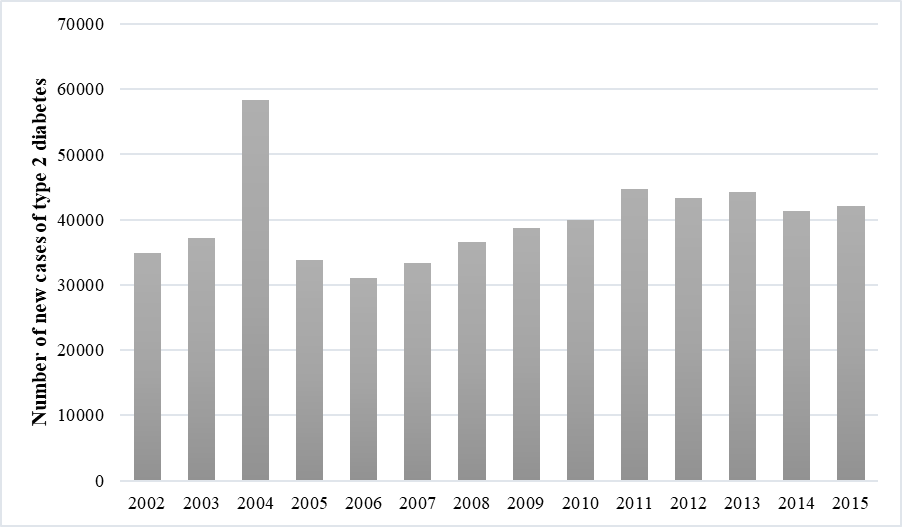

Supplement: S2 Fig — (TIF) [file pmed.1003052.s004.tif]

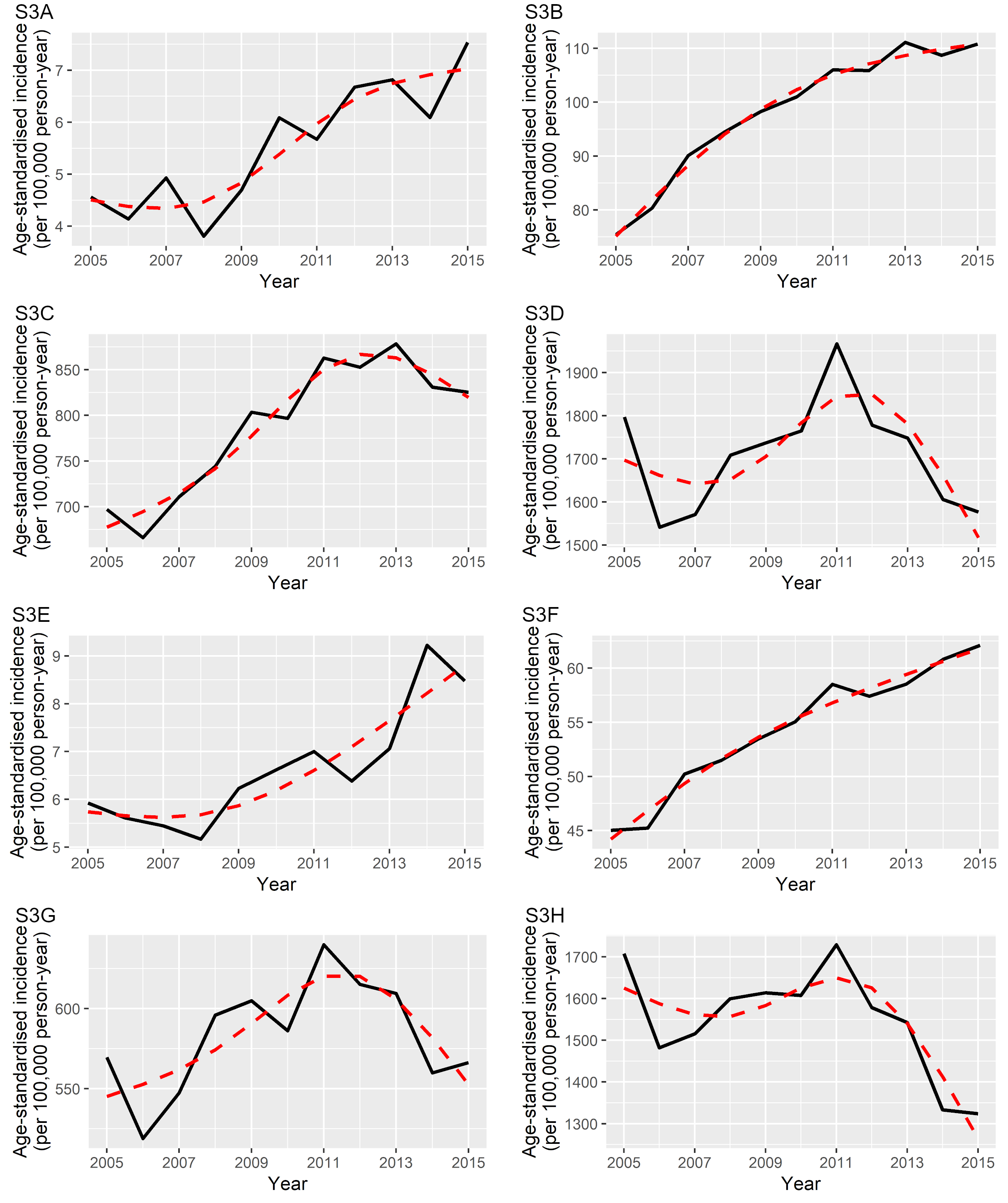

Supplement: S3 Fig — (A) Incidence trends of type 2 diabetes in boys aged <20 years using restricted cubic spline. (B) Incidence trends of type 2 diabetes in men aged 20 to <40 years using restricted cubic spline. (C) Incidence trends of type 2 diabetes in men aged 40 to <60 years using restricted cubic spline. (D) Incidence trends of type 2 diabetes in men aged ≥20 years using restricted cubic spline. (E) Incidence trends of type 2 diabetes in girls aged <20 years using restricted cubic spline. (F) Incidence trends of type 2 diabetes in women aged 20 to <40 years using restricted cubic spline. (G) Incidence trends of type 2 diabetes in women aged 40 to <60 years using restricted cubic spline. (H) Incidence trends of type 2 diabetes in women aged ≥20 years using restricted cubic spline. (TIF) [file pmed.1003052.s005.tif]

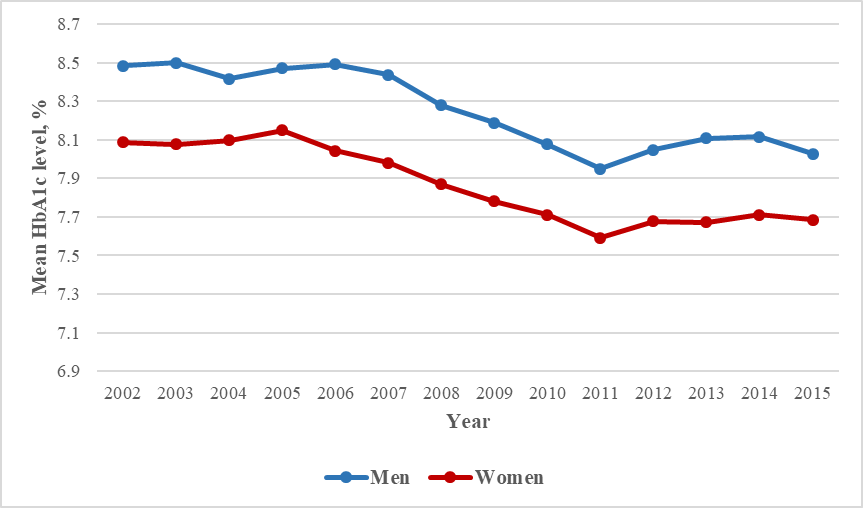

Supplement: S4 Fig — HbA1c, glycated haemoglobin. (TIF) [file pmed.1003052.s006.tif]
