# Supplementary material for: Secular trends in incidence of type 1 and type 2 diabetes in Hong Kong: A retrospective cohort study
Source: PLoS Med. 2020 Feb 20;17(2):e1003052. doi: 10.1371/journal.pmed.1003052 (PMC7032690; doi:10.1371/journal.pmed.1003052)
Supplement: Attachment — Submitted filename: Response to Reviewers_PLOSMED CK.docx [file pmed.1003052.s008.docx]

**Response to Reviewers**

**Editor**

***Comment 1***

Title – I wonder if a clearer study design could be used for the 2nd part of the title?

***Response:***

Thank you for the suggestion. The title is now changed to “Secular trends on incidence of type 1 and type 2 diabetes in Hong Kong”.

***Comment 2***
Abstract – there should be 3 sections: Background, Methods and Findings, Conclusions- please recast.

***Response:***

The abstract organisation has been corrected.

***Comment 3***

Please quantify any results related to data with 95%Cis and p values.

***Response:***

95% confidence interval and p-values have been added to relevant results in the abstract.

***Comment 4***

Please provide a sentence on the limitations of the study as the final sentence of the ‘Methods and Findings’ section.

***Response:***

We have added the following sentence to the end of the “Methods and Findings” section: “The present study is limited by the reliance of electronic medical records for identification of people with diabetes which may result in incomplete capture of diabetes cases. The differentiation of type 1 and type 2 diabetes was based on an algorithm subjected to potential misclassification.”

***Comment 5***

Please do add some summary level demograhic information.

***Response:***

In the revised abstract, we have now provided information on mean age and gender distribution for the cohort.

***Comment 6***
Data – you say that no access is allowed – not even for users who meet requirements?

***Response:***

Thank you for the interest. The investigators made special application to the Hong Kong Hospital Authority to gain access to the clinical data which was granted after careful scrutiny of our request. The clinical data was fully anonymised and not transferrable to third parties in accordance to privacy law and regulation in Hong Kong.

***Comment 7***
At this stage, we ask that you include a short, non-technical Author Summary of your research to make findings accessible to a wide audience that includes both scientists and non-scientists. The Author Summary should immediately follow the Abstract in your revised manuscript. This text is subject to editorial change and should be distinct from the scientific abstract. Please see our author guidelines for more information: <https://journals.plos.org/plosmedicine/s/revising-your-manuscript#loc-author-summary>

***Response:***

An Author Summary has been added.

***Comment 8***

Please use square brackets for refs in the main text.

***Response:***

This has been changed.

***Comment 9***

Line 113 – type 1 or 2?

***Response:***

On line 113 in the original manuscript, it was correct to read “type 1” diabetes. The context was that the previous publication by Quan and colleagues on diabetes incidence in Hong Kong did not differentiate between the two types of diabetes, which has been a major limitation of their study. As highlighted in the discussion section, incidence of type 1 diabetes across age groups spanning youth to older adults has not been adequately reported in Asia previously. In this regard, our results contribute significantly to understanding the contemporary trends in type 1 diabetes in Asia. We have now revised the sentence to better communicate this to the reader.

***Comment 10***

Line 167 – please define diabetes codes

***Response:***

Type 1 diabetes codes were defined as ICD-9 codes 250.x1 or 250.x3, and type 2 diabetes codes were defined as ICD-9 codes 250.x0 or 250.x2. We have added this detail in the revised manuscript.

***Comment 11***
Line 177- define how diabetes is defined – what measure?

***Response:***

Diabetes was ascertained based on one or more of the following qualifying criteria: 1) glycated haemoglobin (HbA1c) ≥6.5 % (48 mmol/mol) in any one available HbA1c measurement (6), 2) fasting plasma glucose (FPG) ≥7.0 mmol/L in any one available FPG measurement (7), 3) prescription of non-insulin anti-hyperglycaemic drugs for at least one day, and/or 4) prescription of insulin for at least 28 days continuously, with or without 5) recording of diagnostic code of diabetes based on International Classification of Diseases, Ninth Revision (ICD-9) code of 250.xx, and/or 6) recording of diagnostic code of diabetes according to the Revised Edition of the International Classification of Primary Care, World Organization of National Colleges, Academics, and Academic Associations of General Practitioners / Family Physicians code T89 or code T90. People who received diagnostic coding of diabetes but did not fulfil any of the laboratory or drug criteria throughout the observation were not considered. This information is located in the sub-section “Study population”.

***Comment 12***
Line 199- I am confused by this sentence. Please clarify ‘Of 778,051 people captured in the HKDSD, we excluded 33,916 people with diabetes codes but not fulfilled other criteria of diabetes.

***Response:***

We apologise for the confusion. In this study, diabetes was defined as either fulfilling laboratory criteria or the continuous use of anti-diabetic drugs, whereas receiving a diagnostic code of diabetes (ICD-9 250.xx or WONCA T89 or T90) was not an absolute requirement. Our rationale for this approach was two-fold: Firstly, not every person with diabetes would have received a code of diabetes in the electronic medical record system because coding in the out-patient setting was optional and was at the discretion of the treating physician. Secondly, it was highly unlikely that a person with diabetes based on coding would not have been prescribed anti-diabetic drug AND never fulfilling laboratory criteria of diabetes for the entire duration of the observation time. We have therefore excluded people with diabetes coding only to minimise misclassifying normal individuals as having diabetes. We have provided a justification in the revised manuscript.

***Comment 13***
Throughout you tend to fall back on describing simply diabetes, as this paper is about type 1 and 2, please be careful to always state which (or if both).

***Response:***

Thank you for this comment. We have gone through the manuscript and have ensured that the type of diabetes is now clear in the appropriate context.

***Comment 14***
The word youth seems a little odd, please instead say people under 20 years of age.

***Response:***

This has been changed.

***Comment 15***
Figure 1 – can I suggest you use different colours instead of 3 variations of blue.

***Response:***

The colours in the figure have been changed to improve clarity.

***Comment 16***
Please use the "Vancouver" style for reference formatting, and see our website for other reference guidelines <https://journals.plos.org/plosmedicine/s/submission-guidelines#loc-references>. For example 6 names and then et al.

***Response:***

This has been changed.

***Comment 17***
Main text – is it possible to provide some information perhaps in table for – adding to one of the current tables – with more participant characteristics, eg BMI, married, smoking etc…

***Response:***

Thank you for this suggestion. In the revised manuscript, we have now included a table in the supporting material (S2 Table) on clinical characteristics of the cohort at diagnosis including mean age, age distribution, sex distribution, mean HbA1c, mean fasting plasma glucose, mean LDL-cholesterol and median triglyceride level. Unfortunately, the existing database does not contain information on socioeconomic status, lifestyle factors (diet, exercise, smoking habits), body mass index and blood pressures, and therefore we have been unable to summarise these characteristics in our table.

***Comment 18***

Please provide a STROBE reporting checklist

***Response:***

A STROBE reporting checklist has been provided.

***Comment 19***
Do you have a prespecified analysis plan, if so please provide as a Supp file and a call out in the methods to it. If no plan exists, please state when the analyses were planned in relation to data analysis.

***Response:***

We do not have a pre-specified analysis plan for this study. The analysis was planned after we have retrieved and curated the database. This is now stated in the Methods section under the subheading “Statistical analysis”.


**Reviewer 1**

***Comment 1***

First, joinpoint analysis needs to be described and its use needs to be justified. It's a fairly obscure method and I had trouble finding literature describing it, but it appears to be a restricted linear spline method. However, while I do like the use of spline models, there are reasons to prefer restricted cubic splines.  And spline methods are a) Well known and b) Implemented in popular software including R and SAS, rather than relying on specialized software.

***Response:***
Thank you for this comment. We agree with the reviewer that there are different methods to analyse trends including restricted cubic spline. As suggested, we have approached trend analysis using restricted cubic spline (Fig 1A-1D for men, Fig 2A-2D for women below). However, we continue to feel that joinpoint analysis (Reference 1) is a reasonable approach as it has been used in previous large-scale surveillance of diabetes incidence (References 2,3) as well as other diseases including cancers (Reference 4). From the point of view of curve fitting, restricted cubic spline is indeed more flexible. On the other hand, we believe that restricted cubic spline is not as suitable in identifying a turning point for change in incidence which is the advantage of joinpoint analysis. In the revised manuscript, we respectfully ask to report results from the joinpoint analysis in the main text and in Table 1 but we have also included results from restricted cubic spline analysis in supporting (S3 Fig).

**Fig 1A.** Incidence trends of type 2 diabetes in boys aged <20 years using restricted cubic spline

**Fig 1B.** Incidence trends of type 2 diabetes in men aged 20-<40 years using restricted cubic spline

**Fig 1C.** Incidence trends of type 2 diabetes in men aged 40-<60 years using restricted cubic spline

**Fig 1D.** Incidence trends of type 2 diabetes in men aged ≥60 years using restricted cubic spline

**Fig 2A.** Incidence trends of type 2 diabetes in girls aged <20 years using restricted cubic spline

**Fig 2B.** Incidence trends of type 2 diabetes in women aged 20-<40 years using restricted cubic spline

**Fig 2C.** Incidence trends of type 2 diabetes in women aged 40-<60 years using restricted cubic spline

**Fig 2D.** Incidence trends of type 2 diabetes in women aged ≥60 years using restricted cubic spline

References:

1. Kim HJ et al. Permutation tests for joinpoint regression with applications to cancer rates. Stat Med 2000; 19:335-351 [correction: 2001;20:655]
2. Geiss LS et al. Prevalence and incidence trends for diagnosed diabetes among adults aged 20 to 79 years, United States, 1980-2012. JAMA 2014; 312:1218-1226
3. Carstensen B et al. The Danish National Diabetes Register: trends in incidence, prevalence and mortality. Diabetologia 2008; 51:2187-2196
4. <https://surveillance.cancer.gov/joinpoint/>

***Comment 2***

Second, the results of the regression analysis don't seem to be included. Are the figures the results of the analysis? Are there other results? What about parameter estimates?

***Response:***

Thank you for your comment. We would like to confirm that the APC and AAPC are calculated using regression analysis and this has been clarified in the revised manuscript.

***Comment 3***
Third, categorizing age is almost surely a mistake. Use age as "number of years".

***Response:***

Thank you for this comment. The primary objective of our study was to describe secular trends in diabetes incidence in children/adolescence, young adults and older adults, which are defined by age categories. We have selected age cut-offs that have also been used in other studies to maximise consistency in reporting. For instance, the SEARCH for Diabetes in Youth Study defined youth as people aged <20 years (Reference 1). A number of studies assessing the burden of diabetes in young adults applied a cut-off of 40 (or 45) years (References 2-6). We believe that these are conditions of special interest to clinicians who are the target audience of this paper.

References:

1. Dabelea D et al. Writing Group for the SEARCH for Diabetes in Youth Study Group. Incidence of diabetes in youth in the United States. JAMA 2007; 296:2716-2724
2. Saydah SH et al. The cardiometabolic risk profile of young adults with diabetes in the U.S. Diabetes Care 2019; xx
3. Geiss LS et al. Prevalence and incidence trends for diagnosed diabetes among adults aged 20 to 79 years, United States, 1980-2012. JAMA 2014; 312:1218-1226
4. Gregg EW et al. Trends in lifetime risk and years of life lost due to diabetes in the USA, 1985-2011: a modelling study. Lancet Diabetes Endocrinol 2014; 2:867-874
5. Pavkov ME et al. Effects of youth-onset type 2 diabetes mellitus on incidence of end-stage renal disease and mortality in young and middle-aged Pima Indians. JAMA 2006; 296:421-426
6. Hillier TA et al. Complications in young adults with early-onset type 2 diabetes. Diabetes Care 2003; 26:2999-3005

***Comment 4***

Line 169 - 173  Something very strange is happening here. Perfect results for under 20 and then really bad results for over 20 make me suspicious that something is going wrong somewhere or, perhaps there are very few people under 20 with type I diabetes, which could lead to overfitting. The authors note that 103 people in HKDR had type I and it seems like about 1/3 were under 20 (although this doesn't seem to be explicitly stated).

***Response:***

Clinically, type 1 diabetes is much more likely to occur among children (34.1% of diabetes cases before age 20 years are type 1, average age of diagnosis of type 1 is 22.7 years in the HKDR validation cohort), whereas type 2 diabetes is much more likely among adults (99.4% of diabetes cases after age 20 are type 2, average age of diagnosis 55.9 years in the HKDR validation cohort). Thus, type 1 diabetes is much easier to identify among children (both epidemiologically and clinically), whereas type 1 diabetes is much more challenging to identify among adults due to its relative rarity. This difficulty is a direct consequence of the striking clinical differences in the age of presentation of these subtypes, and we have added this point to the manuscript.

In a companion publication, we described in detail how we derived and validated the algorithms to identify type 1 and 2 diabetes. This manuscript was separately submitted to a medical research methodology journal and is currently under review (Reference 1). In the validation study, we randomized the HKDR population 2:1 to derivation and validation cohorts to avoid overfitting. The reported performance of the algorithm pertains to the validation cohort only, and this has been clarified in the manuscript. In the validation cohort, we found that the identification of type 1 diabetes was expectedly worse among adults than among children. However, the overall prevalence of type 1 diabetes predicted by the algorithm accurately matched the true prevalence of type 1 diabetes across ages, suggesting that this algorithm could be predictably used to determine the prevalence or incidence of type 1 diabetes at the population level (see Fig 1 below, from Reference 1). We have further acknowledged that the estimates of type 1 diabetes incidence among adults require further confirmation in other studies due to the possibility of misclassification. Finally, the incidence rates of type 1 diabetes among children in our study are comparable to previous estimates using a different paediatric type 1 diabetes registry (Reference 2), supporting the validity of our methods.

References:

1. Ke C, Stukel TA, Luk A, Shah BR, Jha P, Lau E, et al. Development and Validation of Algorithms to Classify Type 1 and 2 Diabetes According to Age at Diagnosis Using Electronic Health Records. BMC Medical Research Methodology. *Under review since July 2019.*
2. Huen KF, Low LC, Wong GW, Tse WW, Yu AC, Lam YY, et al. Epidemiology of diabetes mellitus in children in Hong Kong: the Hong Kong childhood diabetes register. J Pediatr Endocrinol Metab. 2000;13: 297-302.

**Fig 1.** [from reference 1] Prevalence of type 1 diabetes (T1D) by age at diagnosis among all diabetes cases in the validation cohort. Prevalence values are generated using the reference standard (true prevalence, dotted line) and the 6 combination candidate algorithms identified in the derivation cohort. The “*B or D*” algorithm was used for the present study.

**A:** Ratios of type 1 diabetes : type 2 diabetes codes ≥0.5; **B:** Ratios of type 1 diabetes : type 2 diabetes codes ≥4; **C:** Insulin treatment within 90 days of diabetes diagnosis; **D**: Prescription of a combination of short- and long-acting insulin and no non-insulin anti-hyperglycaemic treatment within the first year of diabetes diagnosis.

***Comment 5***
Line 210 and other places.  The authors state that many trends were linear, apparently from testing a model that had year as a linear effect on rate. But the fact that a linear trend is statistically significant does not mean the trend was linear, it just means a straight line is a better fit than a null model. The tables and the graphs show that the trends were not linear at all - they went up and down in a complex pattern.

***Response:***

Thank you for this comment. Fig 2C in the revised manuscript shows the age-standardised incidence of type 2 diabetes in middle-aged (age 40-<60 years) and older adults (age ≥60 years) from 2002 to 2015 in Hong Kong. The complex pattern was due to an acute rise in the number of new diabetes cases observed in 2004 which we believe was related to the Severe Acute Respiratory Syndrome (SARS) epidemic in 2003. In the discussion section, we have provided several explanations connecting SARS to this irregularity in our data. Because of this irregularity, we have restricted the trend (joinpoint) analysis from 2005 onward which is highlighted in the Methods section.

***Comment 6***
Table 1 may not be needed. It seems to duplicate the material from the graphs, which are easier to interpret.  If Table 1 is kept, then the AAPC and the p value should be removed - they are misleading.

***Response:***

Thank you for this suggestion. The original Table 1 has been removed in the revised manuscript.

***Comment 7***
The age categories for the figures don't match the age categories for the table - it's unclear why not, or what was actually included in the model - age as 0-20, 20-40 etc or age as 0-4, 5-9 etc or age as integer or what.

***Response:***

Thank you for this comment. The purpose of Fig 1 was to show the change in incidence of type 2 diabetes across age span over 3 time periods of 2006-2009, 2010-2013, and 2014-2015. We used smaller age intervals (0-4 years, 5-9 years, 10-14 years … etcetera) because we felt that this would allow for a more detailed appraisal of change in diabetes incidence over the age span. In addition, we could narrow down the ages at which the highest incidence of diabetes occurred. However, we fully appreciate that this may cause confusion to the reader and would be willing to modify the figure by using age bands of 0-<20 years, 20-<40 years, 40-<60 years and ≥60 years for consistently if the reviewer feel that it is necessary (Modified Fig 1A and 1B below).

Fig 1. Incidence of type 2 diabetes in men (1A) and women (1B) by age stratified by calendar periods of 2006-2009, 2010-2013 and 2014-2015 in Hong Kong

Fig 1A.

Fig 1B.

**Reviewer 2**

***Comment 1***
Study population line 129.  Please comment in limitations section further about diagnosing diabetes by HbA1c alone in children.  Please see Nowicka P, Santoro N, Liu H et al (2011) Utility of hemoglobin A(1c) for diagnosing prediabetes and diabetes in obese children and adolescents. Diabetes Care 34: 1306-11.

***Response:***

Thank you very much for this information. In the limitation section, we have now addressed the inadequacy of not using 2-hour plasma glucose by OGTT as one of the qualifying criteria for diagnosing diabetes which was particularly important for the paediatric population. We do, however, believe that the number of missing cases would be low because it is unlikely that people with type 2 diabetes based on OGTT alone would not fulfil any other criteria (laboratory or anti-diabetic drugs) throughout the 14-year surveillance time.

***Comment 2***
Study population line 135.  Please comment about why 1 day was chosen, is this sufficient time for a diagnosis of diabetes to be made e.g. revised diagnosis and subsequently drugs quickly stopped?

***Response:***

Thank you for this comment. In our electronic medical record system, the minimum duration of drug prescription is 1 day and in the process of curating our database, we have applied this minimum duration as a condition to identify people on anti-diabetic drug treatment. We acknowledge that it is possible that a person without diabetes be mis-diagnosed as having diabetes and prescribed anti-diabetic drugs over a short term inappropriately. To avoid confusion, we have opted to delete this portion of the sentence in the revised manuscript which we hope is acceptable to the reviewer.

***Comment 3***
Study population line 159.  Please comment specifically in the limitations section about the lack of islet cell antibodies for diagnosing T1DM in children. This is often crucial to distinguish T1 from T2 in children especially in absence of c peptide/insulin and in a population of T1 who may be increasingly overweight.  Please see diagnosistic criteria used for T2DM in this recent study Candler TP, Mahmoud O, Lynn RM et al (2018a) Continuing rise of type 2 diabetes incidence in children and young people in the UK. Diabet Med 35: 737-44.

***Response:***

Thank you for this comment to which we fully agree. We have now addressed this limitation in the revised manuscript.

***Comment 4***
Figure 2 needs revising to better display the rising incidence in <20 year olds.  In the current graph, the scale for this age group is not useful to see any change in trend.  Suggest split figures for >20 and <20 to better show change incidence.  This is a major finding in your study but is not displayed clearly in the results.

***Response:***

Thank you for this suggestion. We have split Figure 2 into two separate figures (Fig 2A and Fig 2B) in the revised manuscript.

***Comment 5***
Line 285.  Please expand further in the discussion as the cause of a reduction in incidence in women >60, can the authors speculate or suggest from other studies a cause for this?

***Response:***

Thank you for this comment. The prevalence of obesity has declined in adult women and was stable in adult men between mid-1990s and the present day, which in turn could account for the decrease in diabetes incidence in older women but not older men. Although men and women were equally exposed to health promotion campaigns, women might be more likely to comply and to adopt a healthy lifestyle compared to men. In the revised manuscript, we have highlighted the possible reason for the observed gender difference in diabetes incidence trends.

***Comment 6***
Line 293.  Please comment further regarding lower HbA1c at diagnosis across the time period 2002-2011 - is this statistically significant i.e. can you provide a p value/statistical test?  Was there a specific public health campaign across this period suggesting people present earlier or a screening programme introduced?  Please speculate as the cause of this further in the discussion.

***Response:***

Thank you for this comment. The decline in HbA1c at diagnosis over time was significant with p-value <0.05 for a linear trend in both men and women. The p-values have been added to the text in the Results section of the revised manuscript.

Screening programmes for diabetes have not been formally introduced in Hong Kong. However, we feel that governmental policies to enhance primary care since 2005 followed by establishment of the Primary Care Office in 2010 (<https://www.fhb.gov.hk/download/press_and_publications/otherinfo/101231_primary_care/e_strategy_doc.pdf>, access date 12 October 2019) and dissemination of diabetes treatment guideline titled “Reference Framework of Diabetes Care” in 2010 (<https://www.fhb.gov.hk/pho/english/resource/files/RF_DM_full.pdf>, access date 12 October 2019) highlighting screening for diabetes in people with risk factors could contribute to earlier diagnosis. The increasing number of out-patient and in-patient attendances to public healthcare facilities also supports increasing opportunities for diabetes screening. According to healthcare utilisation statistics of the Hong Kong Hospital Authority (<http://www3.ha.org.hk/Data/HAStatistics/MajorReport>, access date 12 October 2019), the number of specialist and primary care outpatient attendances increased from 11.2 million in 2008/09 to 13.4 million in 2014/15, and the number of in-patient attendances from 1.3 million to 1.6 million over the same period. In the revised manuscript, we have incorporated our speculations in the discussion section.


**Reviewer 3**

***Comment 1***

The author used national wide data to estimate trends of incidence of diabetes types. They could also look into prevalence of diabetes.

***Response:***

Thank you for this suggestion. We have added a table in the supporting materials on the prevalence of diabetes over time (S1 Table). As the algorithm for the differentiation of type 1 and type 2 diabetes was only applicable for incident cases of diabetes, we were unable to report prevalence of type 1 and type 2 diabetes separately.

***Comment 2***
The authors presented data in adults and younger groups, it would be good to separately present data in adults and young adults and children

***Response:***

Thank you for this suggestion and we agree that this would be useful information. However, due to the small number of people <20 years with incident type 1 or type 2 diabetes (between 14-51 incident cases per year), separating into smaller age groups would make trend analysis difficult to interpret. In light of this, we have decided to keep the same age categories and we hope that this is acceptable to the reviewer.

***Comment 3***
What are key drivers for such trends? The authors might have access to clinical information (e.g. BMI) and other sociodemographic data to investigate these?

***Response:***

Thank you for this comment. Unfortunately, we do not have information on socioeconomic status, family history of diabetes, lifestyle (smoking, diet, exercise) or BMI in our dataset to help understand the observed trends in diabetes incidence. In the discussion, we have alluded to stabilisation of adult obesity rates as well as decrease in cigarettes smoking in Hong Kong as contributary factors for the stable or declining trends in diabetes incidence among older adults (>40 years). In contrast to declining exposure to risk factors in adults, obesity in children and adolescents has been rising over the past 20 years and we believe this to be the major driving force for increasing diabetes incidence in young people.

***Comment 4***
Also it would be useful to provide diabetes incidence projection and possibly to calculate the burden attributable to diabetes (DALY) in this country.

***Response:***

Thank you for this suggestion. We also agree that this would be a very interesting analysis and we are currently working on another manuscript on mortality of people with diabetes which also include analysis on years of life loss.
