# Supplementary material for: Secular trends in incidence of type 1 and type 2 diabetes in Hong Kong: A retrospective cohort study
Source: PLoS Med. 2020 Feb 20;17(2):e1003052. doi: 10.1371/journal.pmed.1003052 (PMC7032690; doi:10.1371/journal.pmed.1003052)
Supplement: Attachment — Submitted filename: Response to Reviewers_PLOSMED_second_revision.docx [file pmed.1003052.s009.docx]

**Response to Editors / Reviewers**

**Editor**

***Comment:***
Title- in order to adhere to PLOS Medicine style guide, your title must contain a study descriptor. Please revise to “Secular trends on incidence of type 1 and type 2 diabetes in Hong Kong: a retrospective cohort study”

***Response:***

The title is revised to “Secular trends in incidence of type 1 and type 2 diabetes in Hong Kong: a retrospective cohort study”.

***Comment:***
Data availability- The Data Availability Statement (DAS) requires revision. For each data source used in your study: a) If the data are freely or publicly available, note this and state the location of the data: within the paper, in Supporting Information files, or in a public repository (include the DOI or accession number). b) If the data are owned by a third party but freely available upon request, please note this and state the owner of the data set and contact information for data requests (web or email address). Note that a study author cannot be the contact person for the data. c) If the data are not freely available, please describe briefly the ethical, legal, or contractual restriction that prevents you from sharing it. Please also include an appropriate contact (web or email address) for inquiries (again, this cannot be a study author).

***Response:***

A Data Availability Statement is included at the end of the manuscript and reads as follows: “The data for this study is provided by the Hong Kong Hospital Authority. Subject to local law and regulation regarding the use and distribution personal data, the database used in the present study cannot be deposited in public data repositories. Data can be applied through the Data Sharing Portal of the Hong Kong Hospital Authority (<https://www3.ha.org.hk/data/DCL/Index/>).”

***Comment:***
Abstract- 95% CI is okay, we do not need the words “confidence interval” as this is a standard term

***Response:***

This is corrected.

***Comment:***
Abstract line 81 should be “subject” not “subjected”

***Response:***

This is corrected.

***Comment:***
Abstract conclusions- both sentences are very similar and there is no indication what is meant by youth, young adults or older adults. Please specify age groups and could it be rephrased to “There was an increase in incidence of both Type 1 and Type 2 diabetes between the age of _ and _ but remained stable in the age group __ ”.

***Response:***

Thank you for pointing this out. The abstract conclusion is now revised to: “There was an increase in incidence of type 2 diabetes in people aged <40 years and stabilisation in people aged ≥40 years. Incidence of type 1 diabetes continued to climb in people aged <20 years but remained constant in other age groups.”

***Comment:***
Author summary please reword “over half is coming from Asia” to “over half of the diabetes population come from Asian countries” or similar

***Response:***

Thank you. The sentence is revised as suggested.

***Comment:***
Author summary- please remove “ calls for preventative actions …” as your study determined the trends and the significance of your findings needs a better explanation

***Response:***

Thank you. This part of the sentence is now removed.

***Comment:***
Introduction- I don’t think we have a Type 2 diabetes pandemic. I appreciate the rates are increasing and there are public health concerns but please tone down.

***Response:***

Thank you. This part of the sentence is now removed.

***Comment:***
Introduction- please change “over-weight” to “overweight”

***Response:***

This is changed.

***Comment:***
Methods- please mention earlier in the methods section whether your study had a prospective analysis plan. If not, please provide reasons

***Response:***

We have highlighted that the analysis was not pre-specified and was planned after obtaining and reviewing the content of the database in the section Study Population under Methods.

***Comment:***
Page 24 Line 414- please remove the word “destined” and consider more appropriate language for a scientific article

***Response:***

The sentence is revised to” people who would develop diabetes were diagnosed progressively earlier …”.

***Comment:***
Page 24 please provide a reference to support “women are generally more receptive…”

***Response:***

A reference is now provided.

***Comment:***
Please ensure you use consistent terminology for ethnicity throughout the text- such as “east Asian” or “Chinese” as they are both used interchangeably.

***Response:***

We referred to “East Asians” in the section describing the incidence of type 2 diabetes among people in Korea and in Taiwan. Since Korean are not Chinese, we would prefer to keep the term East Asians in this sentence if this is acceptable.

***Comment:***
Page 27, please consider revising the limitation section to remove the words “firstly” “fifthly” etc as these are not appropriate for a research article

***Response:***

This is revised.

***Comment:***
Throughout the text, when you say older or younger adults please add the age group referred to for additional clarity

***Response:***

Thank you for the suggestion. We have specify the age groups throughout the manuscript.

***Comment:***

Line 161 – does this need updating? 250.xx

***Response:***

“250.xx” is changed to “250”.

***Comment:***

Line 429 “marked reduction” – please be more specific and give a %decline

***Response:***

Thank you. The sentence is revised to: “Anti-smoking advertising campaigns and government policy to ban smoking in many public areas have resulted in reduction in smoking rates over the past 35 years from 23.3% in 1982 to 10.0% in 2017 in Hong Kong”.

***Comment:***
The STROBE checklist cannot contain page or line numbers as these change during the publication process. Please reference the appropriate paragraphs/sections

Response:

The STROBE checklist has been revised.
